# *Dioscorea* spp.: Bioactive Compounds and Potential for the Treatment of Inflammatory and Metabolic Diseases

**DOI:** 10.3390/molecules28062878

**Published:** 2023-03-22

**Authors:** Zhen Wang, Shengnan Zhao, Siyu Tao, Guige Hou, Fenglan Zhao, Shenpeng Tan, Qingguo Meng

**Affiliations:** 1 Key Laboratory of Molecular Pharmacology and Drug Evaluation, School of Pharmacy, Yantai University, Yantai 264005, China; 2 Physiology Group, Department of Basic and Applied Medical Sciences, Ghent University, 9000 Ghent, Belgium; 3 School of Pharmacy, Binzhou Medical University, Yantai 264003, China

**Keywords:** *Dioscorea*, bioactive compounds, anti-inflammatory activity, metabolic diseases, metabolic disorders

## Abstract

*Dioscorea* spp. belongs to the *Dioscoreaceae* family, known as “yams”, and contains approximately 600 species with a wide distribution. It is a major food source for millions of people in tropical and subtropical regions. *Dioscorea* has great medicinal and therapeutic capabilities and is a potential source of bioactive substances for the prevention and treatment of many diseases. In recent years, increasing attention has been paid to the phytochemicals of *Dioscorea*, such as steroidal saponins, polyphenols, allantoin, and, in particular, polysaccharides and diosgenin. These bioactive compounds possess anti-inflammatory activity and are protective against a variety of inflammatory diseases, such as enteritis, arthritis, dermatitis, acute pancreatitis, and neuroinflammation. In addition, they play an important role in the prevention and treatment of metabolic diseases, including obesity, dyslipidemia, diabetes, and non-alcoholic fatty liver disease. Their mechanisms of action are related to the modulation of a number of key signaling pathways and molecular targets. This review mainly summarizes recent studies on the bioactive compounds of *Dioscorea* and its treatment of inflammatory and metabolic diseases, and highlights the underlying molecular mechanisms. In conclusion, *Dioscorea* is a promising source of bioactive components and has the potential to develop novel natural bioactive compounds for the prevention and treatment of inflammatory and metabolic diseases.

## 1. Introduction

Nowadays, metabolic diseases, a non-communicable disease, are a major health hazard in the modern world and have become a global problem [1]. The epidemic of metabolic disorders, such as obesity, type 2 diabetes mellitus (T2DM), non-alcoholic fatty liver disease (NAFLD), and cardiovascular diseases, poses a great challenge to global human health [2]. Chronic inflammation plays a key role in the initiation, propagation, and progression of metabolic disorders [3]. Therefore, it is necessary to find suitable compounds to control inflammation, reduce metabolic disorders, and thus prevent the progression of related diseases [4]. Due to the limitations of modern drug therapy, such as gastrointestinal discomfort, hypoglycemia, liver and kidney damage, there is an increasing emphasis on finding effective medicine from natural plants (e.g., *Coptis chinensis*, *Embelia ribes*, *Cleome*) to reduce oxidative stress and treat inflammation, diabetes, cardiovascular diseases, and metabolic disorders [5,6,7,8,9].

*Dioscorea* is the largest genus in the *Dioscoreaceae* family, with about 600 species, mainly distributed in tropical and subtropical regions of Southeast Asia, Africa, and Central and South America. *Dioscorea* species are commonly known as yam worldwide, mostly with underground tubers or rhizomes, and are a major food source for millions of people in tropical and subtropical regions [10,11]. It is considered the fourth most important tuber crop after potato, cassava, and sweet potato, contributing about 10% of the world’s root and tuber production [12]. In addition to their importance as traditional starchy staples, various species of *Dioscorea* have been domesticated and widely cultivated for nutritional and medicinal purposes [13]. *Dioscorea* tuber contains secondary metabolites that provide additional health benefits [14]. In fact, *Dioscorea* has good ethnobotanical value. In different ethnic communities and geographical regions, different species of *Dioscorea* are used by local people to treat health problems, such as hemorrhoids, coughs, diabetes, rheumatism, stomach pains, diarrhea, and skin infections [15,16].

*Dioscorea* species contain a large number of bioactive compounds, such as phenols, flavonoids, saponins, anthocyanins, carotenoids, allantoins, and water-soluble polysaccharides [12,17]. Modern research has confirmed that *Dioscorea* has a variety of pharmacological activities, such as improving the cardiovascular system, and regulating immune function, as well as anti-tumour, anti-bacterial, anti-inflammatory, and anti-diabetic activities [18,19,20]. Phytochemicals derived from *Dioscorea*, such as polysaccharides, diosgenin, polyphenols, and allantoin, have been widely used for the treatment of inflammatory and metabolic disorders [21,22].

There have been many reports on the phytochemical and anti-inflammatory activities of *Dioscorea* in the past, as well as studies on the treatment of metabolic diseases, such as obesity and diabetes. However, reported reviews on *Dioscorea* have focused on single species, such as *D. alata*, *D. nipponica* Makino, *D. bulbifera*, or generalized pharmacological activities [17,19,20], and there has not been a comprehensive review on anti-inflammatory activity and the treatment of metabolic diseases of *Dioscorea*, which is not conducive to relevant studies. Therefore, we have mainly selected the research results on the bioactive compounds, anti-inflammatory activity, and treatment of metabolic disorders of *Dioscorea* in the last five years. This is beneficial for the reader to quickly understand the latest research progress of *Dioscorea* and provides an updated reference for researchers to facilitate the development of medicine against inflammatory and metabolic diseases.

## 2. Methodology

**Figure 1:**
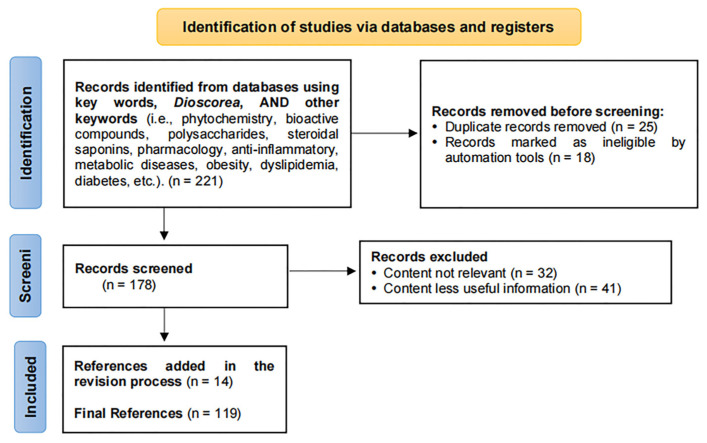
PRISMA flow chart presenting literature data selection.

In order to review the bioactive components and potential to treat inflammatory and metabolic diseases of *Dioscorea*, we searched databases, such as PubMed (https://pubmed.ncbi.nlm.nih.gov, accessed on 17 March 2023), Web of Science (http://apps.webofknowledge.com/, accessed on 17 March 2023), and China National Knowledge Infrastructure (http://www.cnki.net, accessed on 17 March 2023). The literature selection is shown in the PRISM flow chart (Figure 1).

## 3. Bioactive Compounds

**Figure 2:**
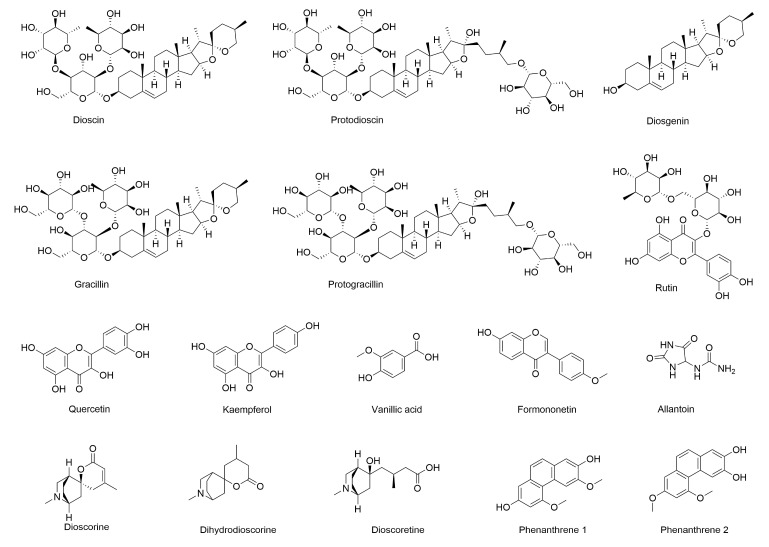
The structures of the main bioactive compounds in *Dioscorea*.

Plant-based secondary metabolites/bioactive compounds play an important role in the prevention of different human diseases [23]. *Dioscorea* is rich in bioactive compounds, such as polysaccharides, steroidal saponins, polyphenols, and allantoin. Figure 2 shows the structures of the main bioactive compounds in *Dioscorea*. It is noteworthy that there are significant differences in the content of bioactive compounds between intra- and interspecies, cultivated and wild varieties [24,25]. The contents of main bioactive compounds of *Dioscorea* are summarized in Table 1.

### 3.1. Polysaccharides

Polysaccharides are the main active component of yam. It is mainly composed of mannose, xylose, arabinose, glucose, and galactose [33]. The various biological activities of yam polysaccharides, such as antioxidant, hypoglycemic, and anti-tumor activities, have attracted the interest of researchers [34,35]. Currently, some yam polysaccharides have been isolated and purified. However, different yam species and different extraction methods yielded polysaccharides with different structures and biological activities [36]. Different concentrations of yam polysaccharides and their derivatives purified by hot water extraction and column chromatography had the same ability to scavenge hydroxyl radicals as Vitamin C. Additionally, the antioxidant activity of the polysaccharides and its derivatives against superoxide anion and lipid peroxidation reached the level of Vitamin C [37]. In addition, three polysaccharides purified from Chinese Huaishan yams had the ability to inhibit α-glucosidase and the relative value-added rate of B16 mouse melanoma cells [38].

The water-soluble polysaccharides (WSP) of *Dioscorea hispida* extracted by aqueous extraction, papain-assisted extraction, and tempeh inoculum-assisted extraction had hypoglycemic effects. Among them, tempeh inoculum-assisted WSP extract had the highest hypoglycemic activity, while papain-assisted WSP extract had the highest glucose absorption inhibition [39]. Another study used hot water extraction, acid extraction, hot-compressed water extraction, and enzyme-assisted extraction of polysaccharides from the peel of the *Dioscorea opposita* Thunb. The polysaccharide yields, hypoglycemic activity, and antioxidant activity of these four extraction methods were compared. It was shown that hot-compressed water extraction and acid extraction gave the highest polysaccharide yields, and acid extraction showed the highest antioxidant activity and α-amylase inhibitory activity [34]. Optimization of the ultrafiltration-assisted extraction of yam polysaccharides using response surface methodology exhibited not only antioxidant activity but also dose-dependent inhibition of proliferation of human gastric gland cancer cells in vitro [40].

A new study shows that the heteropolysaccharide (DOP-2) isolated from *Dioscorea opposita* can modulate the metabolic disorders caused by benzopyrene (BaP). DOP-2 can improve BaP-induced liver injury by enhancing the activity of immune and antioxidant enzymes, exhibiting hepatoprotective activity. Moreover, DOP-2 can modulate the concentration of organic acids to increase the fecal absorption of BaP [41].

### 3.2. Steroidal Saponins

Steroidal saponins are important secondary metabolites of *Dioscorea*. Currently, more than 100 steroidal saponins have been isolated from various *Dioscorea* species, such as stigmastanol, furostanol, spirostanol, and cholestanol [42]. The major saponins are dioscin, gracillin (spirostanol glyclosides), protodioscin, and protogracillin (furostanol glycosides) [26]. Hydrolysis of diosgenin derivatives yields diosgenin, which are an important source of corticosteroids, anti-inflammatory, androgen, estrogen, and contraceptive drugs in the pharmaceutical industry [43,44]. Diosgenin are in high demand in the market. There are 137 species of *Dioscorea*. containing diosgenin, of which 41 species contain more than 1% diosgenin and have great utilization value [45]. More and more studies show that steroidal saponins have a wide range of biological activities, such as anticancer [46,47], antifungal [48], anti-inflammatory [49,50], hypolipidemic [51], and cardioprotective [52]. Lee et al. [53] isolated 15 steroidal saponins from tubers and leaves of *Dioscorea esculenta*, including 4 furanosteroid glycosides and 11 spirostanol glycosides. More importantly, four novel steroidal saponins were identified. A study analyzed the total saponin content of 1151 materials of six yam cultivars and the powder of five wild yam (*D. villosa*). The results showed that the total saponin content ranged from 37.36–129.97 mg/g. The average total saponin content was 42.15 mg/g for *D. cayenensis*, 17.65 mg/g for *D. esculenta*, and 17.44 mg/g for *D. rotundata*, with *D. cayenensis* having the highest total saponin content (78.31 mg/g). No steroidal saponins were found in the other three cultivars (*D. alata*, *D. bulbifera*, and *D. dumetorum*) [29].

Nazir et al. [54] reported for the first time the effective extraction method, solvent system, drying process and seasonal variation of diosgenin content of *Dioscorea deltoidea*. Drying the plant material in a cool place was the best drying method with the highest diosgenin content of 1.43% dry weight (DW). Microwave-assisted extraction with 50% ethanol yielded the highest amount of diosgenin in a short time (within 3 min) The tubers collected in December had the highest diosgenin content of 1.19% DW. In addition, latitude, annual parity temperature, and maximum annual precipitation affected steroid saponin biosynthesis. RDA analysis showed that environmental factors limited the total variance of steroid saponin biosynthesis (phytosterols, diosgenin, and steroid saponins) by 64.67% [55].

### 3.3. Polyphenols

Polyphenols, as secondary metabolites of plants, are a class of phytochemicals with potential health-promoting effects. Polyphenols are classified into flavonoids and non-flavonoids (phenolic acids) [56]. Zhang et al. [57] used high performance liquid chromatography–high resolution electrospray ionization mass spectrometry to identify seven phenolic compounds extracted from purple yam (*Dioscorea alata* L). This study not only confirmed the presence of ferulic and mustard acids, but also identified previously unidentified phenolic compounds, including vanillic acid, caffeic acid, *p*-coumaric acid, kaempferol, and quercetin hydrate. In addition, gallic acid, rutin and quercetin were confirmed to be present in the leaf extract of *Dioscorea pentaphylla* [58]. Jing et al. [59] isolated 13 monocyclic phenols and two flavonoids (formononetin and (+)-Catechin) from *D. collettii*. Among them, two benzylacetone derivatives, three phenylpropanoids, and formononetin were isolated for the first time from the genus *Dioscorea*.

Two flavonols, caryatin and 3′-*O*-methyl caryatin, isolated from the tubers of *D. nummularia* accounted for more than 92% of the stable methanolic extract by Fel et al. [28]. This study further determined the content of these two flavonols in the tubers and bulbils of 411 local varieties and hybrids of eight edible yam species. The results showed that these substances varied considerably at intra- and interspecific levels. Among them, caryatin in *D. bulbifera*, *D. alata*, and *D. nummularia* accounted for more than 90% of the total antioxidant activity of the methanolic extract of the tubers. Du et al. [60] isolated two new phenols and six known phenols from *Dioscorea zingiberensis*. One of the known phenols with a trihydroxy-diarylheptane backbone could mediate mitochondrial dysfunction by reducing ATP depletion and reactive oxygen species production, thereby preventing sodium taurocholate-induced pancreatic acinar cells necrosis. Zhou et al. [61] isolated and identified 17 phenolic compounds from *Dioscorea* leaves. The major phenolic compound was rosmarinic acid with the highest content of 22.31 ± 1.33 mg/g DW in the leaves of *Dioscorea glabra* Roxb. Rutin was the major flavonoid, followed by quercetin. Rutin was highest in the leaves of *Dioscorea alata*, with 8.66 ± 0.29 mg/g DW. Leaf extracts showed good antioxidant activity against hydrogen peroxide-induced oxidative stress.

### 3.4. Allantoin

Allantoin is one of the secondary metabolites of yam. Allantoin has antidiabetic and antioxidant activity and promotes wound healing [17]. Lebot et al. [29] detected six cultivars (*D. alata*, *D. bulbifera*, *D. dumetorum*, *D. cayenensis*, *D. esculenta*, and *D. rotundata*) for allantoin content ranging from 0.23 to 22.35 mg/g DW. A study on age-specific differences in *Dioscorea polystachya* showed that four-year-old tubers with the highest allantoin content were the best choice for industrial production of large quantities of allantoin [62]. Studies have shown that allantoin derived from *Dioscorea batatas* significantly reduced body weight and plasma biochemical parameters, and inhibited structural damage to the liver, pancreas, and skeletal muscle in mice with high-fat-diet (HFD)- and streptozotocin (STZ)-induced diabetes [63].

### 3.5. Alkaloids

Alkaloids are the largest group of secondary metabolites, consisting of nitrogen bases synthesized from amino acids. The alkaloids present in *Dioscorea* tuber are dioscorine, dihydrodioscorine, dioscoretine, and dumetorine [64,65]. The content of alkaloids in *D. belophylla* was 0.68 mg/100 g [30]. *D. alata* and *D. esculenta* contained significant amounts of alkaloids at 1.64 mg/100 g and 1.89 mg/100 g, respectively [31]. Another study showed that the alkaloid content in different *Dioscorea* species varied from 7.2 to 16 mg/100 g [24]. Alkaloid derivatives have analgesic, antitussive, and antibacterial pharmacological activities [12]. However, one of the major alkaloids in yam, dioscorine, is a toxic isoquinoline alkaloid. Dioscorine can be effectively removed by soaking in 1.0 M sodium chloride solution for 5 days compared to the traditional detoxification method of peeling and slicing and soaking in running river water for seven days [17,66]. In addition, Haji et al. [67] designed a detoxification device based on a water cycle operation and found that a rotating operation of the water cycle was more effective in removing doscorine.

### 3.6. Phenanthrene Derivatives

Phenanthrene is a group of polycyclic aromatic hydrocarbons naturally occurring in various plants. Kim et al. [32] quantified the contents of three phenanthrenes 2,7-dihydroxy-4,6-dimethoxyphenanthrene, 6,7-dihydroxy-2,4-dimethoxyphenanthrene, and batatasin in the pericarp of *D. batatas*, *D. polystachya*, *D. quinqueloba*, and *D. bulbifera*. The highest content of 2,7-dihydroxy-4,6-dimethoxyphenanthrene was 173.69 μg/g in *D. quinqueloba*. *D. polystachya* was rich in 6,7-dihydroxy-2,4-dimethoxyphenanthrene and batatasin at 166.99 μg/g and 419.73 μg/g, respectively. It has been shown that 2,7-dihydroxy-4,6-dimethoxyphenanthrene isolated from *D. batatas* Decne peel extract has antioxidant and anti-inflammatory activities [68]. In addition, 2,7-dihydroxy-4,6-dimethoxyphenanthrene isolated from *D. opposita* and *D. batatas* Decne peel extracts had protective properties against intestinal mucosal damage and inhibited lipopolysaccharide (LPS)-induced inflammatory responses in BV2 microglia [69,70]. Phenanthrene and dihydrophenanthrene derivatives isolated from *D. communis* were shown to possess anticholinesterase and antioxidant activities [71].

## 4. Anti-Inflammatory Activity

Chronic inflammatory diseases are recognized as the most important cause of death, with over 50% of deaths attributable to inflammation-related diseases, such as ischemic heart disease, cancer, diabetes, and NAFLD [72]. The bioactive compounds of *Dioscorea*, such as phenanthrene derivatives, steroidal saponins, polysaccharides and polyphenols, exert anti-inflammatory activity and have excellent effects on a wide range of inflammatory diseases [68,73,74].

### 4.1. Enteritis

Yam polysaccharides are not digested by gastrointestinal fluids. Most of the indigestible yam polysaccharides are degraded and utilized by intestinal microorganisms, promoting the production of short-chain fatty acids (SCFAs) and the relative abundance of probiotic bacteria [75]. In LPS-stimulated cocultured Caco-2/Raw264.7 cells, both yam polysaccharides and fecal fermented yam polysaccharides significantly inhibited the levels of inflammatory mediators nitrogen oxide (NO)/Inducible nitric oxide synthase (iNOS), tumor necrosis factor-α (TNF-α) and interleukin-1 beta (IL-1β), and increased the expression of intestinal tight junction protein 1 (ZO-1) and Occludin. Among them, the anti-inflammatory and intestinal barrier-protective effects of fecal fermented yam polysaccharides were superior to those of yam polysaccharides [74].

Chen et al. [76] reported for the first time that anthocyanins (DACNs) isolated from *Dioscorea alata* L. tubers exhibited anti-inflammatory activity in a trinitrobenzene sulfonic acid (TNBS)-induced colitis model in mice. Compared with the TNBS group, 80 mg kg^−1^ DACNs significantly reduced disease scores in mice with inflammatory bowel disease (IBD). DACNs significantly promoted the expression of colonic tight junction-related proteins. Similarly, DACNs significantly reduced the concentrations of myeloperoxidase and iNOS in colonic tissues, as well as TNF-α and interferon-γ (IFN-γ) in serum. It was shown that *Dioscorea batatas* peel extract not only reduced nitric oxide production and expression of pro-inflammatory proteins in LPS-stimulated RAW264.7 cells, but also decreased the level of reactive oxygen species in hydrogen peroxide-stimulated HCT116 cells. Moreover, yam bark extract promoted nuclear factor(erythroid-derived 2)-like 2 (Nrf2)-mediated antioxidant enzyme expression and inhibited nuclear factor kappa B (NF-κB)-mediated inflammatory responses in macrophages, thereby attenuating dextran sodium sulfate (DSS)-induced acute colitis in mice [77]. 6,7-Dihydroxy-2,4-dimethoxyphenanthrene (CYP4) isolated and purified from Chinese yam peel could inhibit extracellular signal-regulated kinase 1/2 (ERK1/2), NF-κB p65, pNF-κB, and cyclooxygenase-2 (COX-2) expression in colon tissue to inhibit DSS-induced intestinal mucosal injury in mice. It was shown that the daily intake of 120 mg/(kg·d) of Chinese yam peel extract and 4 mg/(kg·d) of CYP4 had good preventive effect on acute colitis in mice [69].

### 4.2. Arthritis

Gouty arthritis (GA) is a very common form of inflammatory arthritis. Controlling inflammation is the key to preventing gouty arthritis [78]. NOD-, LRR-, and pyrin-domain containing protein 3 (NLRP3) inflammatory vesicles induce inflammation and pyrophosphate cell death in response to pathogens and endogenous activators. Excessive activation of NLRP3 inflammatory vesicles leads to the development of inflammatory diseases and cancer [79]. In vitro and in vivo studies have shown that dioscin has anti-inflammatory effects on sodium urate (MSU)-mediated gouty arthritis. The underlying molecular mechanism may be that dioscin reduces inflammation by reducing the production of pro-inflammatory cytokines and inhibiting the activation of inflammasome NLRP3 and Toll-like receptor 4 (TLR 4)/NF-κB signaling pathway [80]. In addition, in vivo studies in mice with GA found that total yam saponins, the main components of which are dioscin, protodioscin, and pseudo protodioscin, exert anti-inflammatory effects for GA by regulating the expression of some key genes in the neutrophilic alkaline phosphatase-3 (NALP3), caspase-1, apoptosis-associated speck-like (ASC), and mitogen-activated protein kinase (MAPK)-peroxisome proliferator-activated receptor γ (PPARγ) signaling pathways [81,82]. Diosgenin from *Dioscorea nipponica* Makino had a therapeutic effect on collagen-induced arthritis in mice. *Dioscorea* exhibited anti-arthritic activity by down-regulating the differentiation of Th17 cells, significantly reducing the Th3 cell ratio in CD17+ T cells, IL-17 and IL-6 levels, and RORat expression [83].

### 4.3. Dermatitis

Natural saponins have anti-inflammatory effects. Jegal et al. [84] investigated the effect of Gracillin, the main saponin isolated from the rhizome of *Dioscorea quinqueloba*, on atopic dermatitis (AD). It was found that in 2,4-dinitrochlorobenzene (DNCB)-induced AD mice, gracillin effectively alleviated AD symptoms (redness, itchiness, swelling, and skin lichenification). In addition, the impaired skin barrier and skin hydration were significantly improved. Gracillin also inhibited the overproduction of IL-4 in vitro and in vivo. In addition to AD, paeonol, the bioactive phenol in *Dioscorea japonica* Thunb., plays an effective role in dry skin diseases. In an acetone–ether–water (AEW)-treated mouse model of dry skin, paeonol attenuated skin inflammatory responses and scratching behavior and reduced CXCR3-driven expression of spinal astrocyte activity-dependent genes [85].

### 4.4. Acute Pancreatitis

Acute pancreatitis (AP) is one of the common causes of hospitalization for gastrointestinal diseases and has a very high morbidity and mortality rate [86]. Endoplasmic reticulum stress (ERS) and Gasdermin D (GSDMD) affect AP. GSDMD accumulates in the endoplasmic reticulum of the glandular alveolar cells, which exacerbates the local pancreatic symptoms and systemic inflammation of AP. The mechanism of action of diosgenin derivative on AP was investigated by L-arginine-induced AP mouse model and mouse pancreatic alveolar cell model in vitro. It was shown that diosgenin derivatives isolated from *Dioscorea zingiberensis* could act as GSDMD inhibitors and inhibit L-arginine-induced AP by regulating GSDMD in the endoplasmic reticulum through the thioredoxin-interacting protein (TXNIP)/hypoxia-inducible factor-1α (HIF-1α) pathway [87]. Moreover, diosgenin were protective against pancreatic follicular cell injury in vivo and in vitro. Dihydrodiosgenin (5 or 10 mg/kg) had inhibitory effects on Tauro-induced AP, caerulein-induced AP, and palmitoleic acid plus ethanol-induced AP. The potential mechanism of action is to prevent excessive inflammatory responses by restoring mitochondrial function in the pancreas and inhibiting the phosphoinositide 3-kinase gamma (PI3Kγ)/protein kinase B (Akt) pathway. More importantly, this ameliorated acute lung injury associated with pancreatitis [88].

### 4.5. Neuroinflammation

Neuroinflammation is caused by endogenous or exogenous injury activating microglia. *Dioscorea nipponica* rhizome ethanol extract (DNRE) and dioscin significantly inhibited LPS-induced microglia activation and reduced the levels of pro-inflammatory factors (IL-1β, IL-6) and inflammatory enzymes (iNOS, COX-2). The dioscin of DNRE may be an inhibitor of p65 phosphorylation and translocation, and inhibition of transcription of multiple inflammatory cytokines and has a preventive effect on neuroinflammation [89,90]. Another study showed that 6,7-dihydroxy-2,4-dimethoxyphenanthrene (DHDMP), a phenanthrene compound isolated from *Dioscorea batatas* Decne, also significantly reduced the production of pro-inflammatory mediators and inhibited nuclear translocation of NF-κB and phosphorylation of p38 MAPK in BV2 cells [70].

Overall, these studies support the anti-inflammatory activity of *Dioscorea* extracts against enteritis, arthritis, dermatitis, pancreatitis, and neuroinflammation. The bioactive substances exerting anti-inflammatory effects are mainly yam polysaccharides, polyphenols, phenanthrene derivatives, and steroidal saponin derivatives. The anti-inflammatory mechanism is mainly inhibition of pro-inflammatory cytokine production and modulation of inflammation-related signaling pathways.

## 5. Prevention and Treatment of Metabolic Diseases

**Figure 3:**
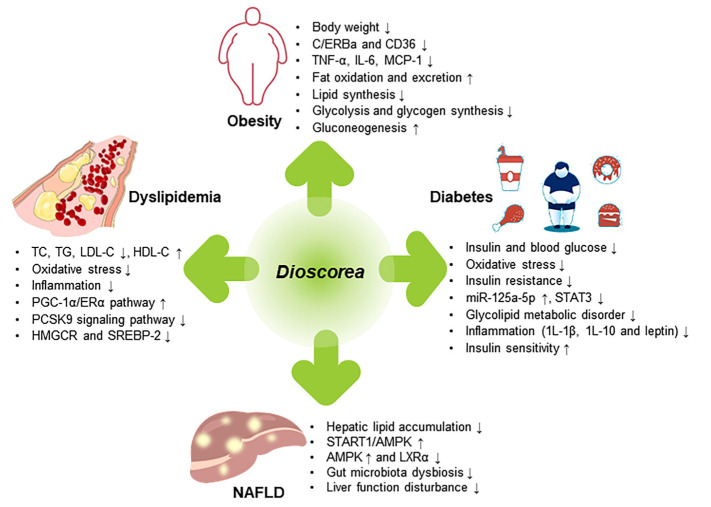
The therapeutic effects of *Dioscorea* on metabolic diseases.

Metabolic diseases, such as obesity, T2DM, dyslipidemia, and NAFLD, are a serious threat to human health. The search for natural active compounds of plant origin to prevent and treat metabolic diseases is a hot research topic today. A growing number of studies have shown that the bioactive compounds of *Dioscorea* play an important role in the prevention and treatment of metabolic diseases (Table 2). The therapeutic effects of *Dioscorea* on metabolic diseases are shown in Figure 3.

### 5.1. Obesity

Obesity is a chronic multisystem disease that leads to a reduced life expectancy, reduced quality of life, and disability, especially for people with cardiovascular disease, diabetes, and cancer [107,108]. It was shown that the addition of *Dioscorea batatas* extract to the diet for 14 weeks significantly inhibited HFD-induced weight gain in mice. Supplementation with yam extract inhibited the expression of adipogenic transcription factor and its target gene cluster of differentiation 36 (CD36), reducing visceral fat accumulation and suppressing HFD-induced obesity. In addition, yam extract reduced the levels of pro-inflammatory cytokines (TNF-α, IL-6, monocyte chemoattractant protein-1 (MCP-1)) in HFD-induced adipose tissue [91]. *Dioscorea japonica* propagules promote fat oxidation and utilization and inhibits cholesterol synthesis. For carbohydrate metabolism, the propagules inhibit glycolysis and glycogen synthesis and promote gluconeogenesis. In summary, the propagules regulate fat metabolism and carbohydrate metabolism to inhibit disorders of carbohydrate and fat metabolism in high fat-loaded mice [92].

Jeong et al. [93] reported for the first time the anti-obesity effect of the *n*-butanol extract of *D. oppositifolia*. The main components of the extract were 3,5-dimethoxy-2,7-phenanthrenediol and (3R,5R)-3,5-dihydroxy-1,7-bis(4-hydroxyphenyl)-3,5-heptanediol. The results showed that the extract inhibited dietary fat absorption and increased fecal fat excretion to reduce body weight, and adipose tissue weight in HFD-induced obese mice. Du et al. [94] reported metabolic studies on the anti-obesity effects of diarylheptanoid, isolated and identified from *Dioscorea zingiberensis*. Diarylheptanoid inhibits differentiation and fat accumulation in 3T3-L1 cells by balancing energy metabolism and regulating lipid metabolism. This involves multiple aspects of metabolism, including regulation of tricarboxylic acid (TCA) cycle and glycolysis, disruption of amino acid metabolism, inhibition of purine metabolism, and reduction of ab initio synthesis of fatty acids and lipids.

### 5.2. Dyslipidemia

Dyslipidemia is an imbalance of plasma lipids, such as total cholesterol (TC), triglyceride (TG), low-density lipoprotein (LDL), and high-density lipoprotein (HDL), caused by abnormal lipid metabolism, which may lead to obesity, diabetes, and cardiovascular disease [109]. A study showed that resistant starch (RS) from purple yam (*Dioscorea alata* L.) reduced body weight, liver weight, and adipose tissue weight in hyperlipidemia-induced hamsters. RS improved lipid composition and regulated lipid metabolism by decreasing TC, TG, and LDL-C concentrations and increasing HDL-C concentrations [95]. In addition, Chinese yam polysaccharides also have anti-hyperlipidemic activity and can significantly reduce LDL and TC levels [96]. Dioscin, diosgenin and pseudoprotodioscin are phytoestrogens that are structurally similar to estrogens, with antioxidant, anti-inflammatory, anti-adipogenic, and inhibitory effects on atherosclerosis associated with estrogen deficiency [97,98,110]. Dioscin was found to not only regulate lipid metabolic homeostasis, but also attenuate postmenopausal atherosclerosis in HFD and ovariectomy (HFD-OVX)-induced low-density lipoprotein receptor-deficient (LDLR-/-) mice by inhibiting oxidative stress, inflammation, and apoptosis in part dependent on the peroxisome proliferator-activated receptor-γ coactivator 1-α (PGC-1α)/estrogen receptor alpha (Erα) pathway [98].

The Di’ao Xinxuekang (DXXK), extracted from *Dioscorea nipponica* Makino, attenuated HFD-induced hyperlipidemia and atherogenesis in mice, probably through inhibition of the proprotein convertase subtilisin/kexin type 9 (PCSK9) signaling pathway [99]. Shi et al. [100] reported that Shuangyu Tiaozhi granules (STG), consisting of two herbs from the genus *Dioscorea*, reduced hypercholesterolemia and hepatic cholesterol accumulation. 3-Hydroxy-3-methylglutaryl-CoA reductase (HMGCR) is the rate-limiting enzyme in a key step of catalytic cholesterol synthesis. STG inhibits the expression of HMGCR and sterol regulatory element-binding protein-2 (SREBP-2) and increases plasma LDL-C clearance to lower cholesterol levels, thereby slowing the onset and progression of coronary heart disease.

### 5.3. Diabetes

Diabetes is a metabolic disease characterized by high blood glucose. The prevalence of diabetes is 10.5% (536.6 million people) in 2021 and is expected to rise to 12.2% in 2045, by which time it will affect 783.2 million people worldwide [111,112]. T2DM is the most prevalent metabolic disorder characterized by insulin resistance, which results from chronic hyperglycemia and inadequate response of peripheral tissues to circulating insulin [113]. *Dioscorea* extracts for the treatment of diabetes and its complications, including diabetic nephropathy, diabetic liver disease, diabetic neuropathy, diabetic vasculopathy, diabetic reproductive dysfunction, diabetic eye disease, and diabetic hearing impairment, have been widely reported [63,114,115,116,117]. *Dioscorea esculenta* administration for eight weeks was reported to significantly inhibit the increase in plasma insulin and fasting glucose levels in T2DM rats. In addition, metabolic insulin-sensitive glucose metabolic clearance was increased, and insulin resistance was improved [101]. A novel *Dioscorea opposita* Thunb polysaccharide-zinc inclusion complex was shown to reduce blood glucose and insulin levels, as well as improve lipid levels and oxidative stress in STZ-induced diabetic rats [102].

A study has shown that dioscin from *Dioscoreae nipponicae* rhizomes improves insulin resistance by reducing hyperglycemia and hyperlipidemia. Dioscin promotes glycogen synthesis and inhibits gluconeogenesis and lipogenesis by modulating the miR-125a-5p/STAT3 signaling pathway, thus significantly alleviating the glucolipid metabolism disorders of T2DM [103]. Chinese yam polysaccharides reduced body weight in obese mice. Additionally, it improved insulin resistance by reducing serum inflammatory factors 1L-1β, 1L-10, and leptin and increasing insulin sensitivity [96]. *Dioscorea* and its extracts have the potential to become functional foods and drugs for the treatment of T2DM.

### 5.4. Non-Alcoholic Fatty Liver Disease

NAFLD is a metabolic stress liver injury that is closely associated with obesity, insulin resistance, T2DM, atherosclerosis, and metabolic syndrome, and is a major cause of cirrhosis and hepatocellular carcinoma [118,119]. Dioscin significantly attenuated hepatic lipid accumulation and improved serum and hepatic biochemical index levels in vitro and in vivo [98,103]. Dioscin modulates the expression levels of downstream proteins, including promoting the expression of silent information regulator of transcription 1 (SIRT1), AMP-activated protein kinase (AMPK), carnitine palmitoyltransferase (CPT), forkhead box O 1 (FoxO1), and adipose triglyceride lipase (ATGL), and inhibiting the expression of SREBP-1c, FAS, and SCD to suppress lipid metabolism. It was shown that dioscin significantly improves hepatic lipid metabolism and reduces TG accumulation to prevent NAFLD by activating the START1/AMPK signaling pathway [104]. Diosgenin activates the AMPK pathway and inhibits LXRα to suppress high glucose-induced TG accumulation, thereby preventing hepatic lipid accumulation in vitro and in vivo. Diosgenin also improved HFD-induced liver function disturbance [105]. In addition, fecal metabolomics analysis showed that diosgenin improved dysbiosis of gut microbiota HFD-induced NAFLD rats, which was significantly associated with lipid and amino acid metabolism [106].

In short, *Dioscorea* extracts, such as polyphenols, polysaccharides, resistant starch, dioscin, and diosgenin elements, have excellent preventive and therapeutic effects on obesity, dyslipidemia, diabetes, and NAFLD. Bioactive extracts of *Dioscorea* ameliorate metabolic disorders by modulating relevant signaling pathways and molecular targets, thus inhibiting the damage of metabolic diseases.

## 6. Conclusions

*Dioscorea* is not only the main source of food, but also a potential source of bioactive compounds. *Dioscorea* is rich in beneficial bioactive compounds, such as yam polysaccharides, diosgenin, dioscin, allantoin, and polyphenols. A growing number of in vitro and in vivo studies have demonstrated the utility of these bioactive compounds in the prevention and treatment of inflammation, obesity, diabetes, dyslipidemia, and NAFLD. Various signaling pathways associated with oxidative stress and inflammation, such as NF-κB, NLRP3, MAPK-PPARγ, PI3Kγ/Akt, PGC-1α/Erα, and START1/AMPK, are the main targets of action of the bioactive compounds. In summary, *Dioscorea* is a promising medicinal and edible plant, which deserves more intensive research. In the future, the isolation and identification of natural bioactive compounds of *Dioscorea* should be considered. Moreover, it is necessary to investigate the role and mechanism of these natural bioactive compounds in anti-inflammatory and preventive metabolic diseases. As a medicinal and edible plant, *Dioscorea* has the potential to be developed into functional foods for the prevention and treatment of inflammatory and metabolic diseases.

## Figures and Tables

**Table 1:** The contents of main bioactive compounds in *Dioscorea*.

Compounds	Content	Specie	Plant Part	Ref.
Protodioscin	13.5–14.9 mg/g	*D. nipponica*		[26]
Protogracillin	7.7–8.4 mg/g		Tuber	
Dioscin	2.3–3.8 mg/g			
Gracillin	0.7–1.2 mg/g			
Gallic acid	1.34–2.35 mg/g DW	*D. alata*	Tubers and bulbils	[17]
Epicatechin	0.45–10.71 mg/g DW	*D. alata*	Tubers and bulbils	[17]
Catechins	25.18 mg/g	*D. bulbifera*	Bulbils	[27]
	6.96 mg/g	*D. bulbifera*	Tubers	
	0.32 mg/g	*D. esculenta*	Tubers	
Phenolic acids	4.33 mg/g	*D. bulbifera*	Tubers	
	4.87 mg/g	*D. alata*	Tubers	
	9.55 mg/g	*D. nummularia*	Tubers	
Caryatin	1030 µg/g DW	*D. alata*, *D. bulbifera*, *D. cayenensis*, *D. dumetorumacc*, *D. esculentaacc*, *D. nummularia acc*, *D. pentaphylla*	Tubers	[28]
3′-*O*-Methyl caryatin	457 µg/g DW			
Allantoin	4.23–20.8 mg/g	*D. alata*, *D. bulbifera*, *D. cayenensis*, *D. dumetorum*, *D. esculenta*, *D. rotundata*	Powders	[29]
	0.68 mg/100 g	*D. belophylla*	Tubers	[30]
	1.64 mg/100 g	*D. alata*	Tubers	[31]
	1.89 mg/100 g	*D. esculenta*	Tubers	[31]
Alkaloid	7.2–16 mg/100 g DW	*D. oppositifolia*, *D. hamiltonii*, *D. pubera*, *D. wallichii*, *D. hispida*, *D. pentaphylla*, *D. bulbifera*, *D. glabra*, *D. alata*	Tubers	[24]
2,7-Dihydroxy-4,6-dimethoxyphenanthrene	9.79–173.69 μg/g	*D. batatas*, *D. polystachya*, *D. quinqueloba*, *D. bulbifera*	Peels	[32]
6,7-Dihydroxy-2,4-dimethoxyphenanthrene	46.65–166.99 μg/g	*D. batatas*, *D. polystachya*		
Batatasin	97.19–419.73 μg/g	*D. batatas*, *D. polystachya*		

DW: dry weight.

**Table 2:** The therapeutic effects of *Dioscorea* on specific metabolic diseases.

Species	Metabolic Diseases	Study Type	Main Results	Bioactive Compounds	Ref.
*D. batatas* rhizome	Obesity	HFD-induced mice	Downregulated the adipogenic transcription factor and its target gene (CD36) Decreased the expression of proinflammatory cytokines (TNF-α, MCP-1, and IL-6)	-	[91]
*D. Japonica* propagules	Obesity	High-fat-loaded mice	Suppressed carbohydrate and fat metabolism disorders	-	[92]
*D. oppositifolia* rhizomes	Obesity	HFD-induced obese mice	Suppressed feeding efficiency and fat absorption	3,5-dimethoxy-2,7-phenanthrenediol (3R,5R)-3,5-dihydroxy-1,7-bis(4-hydroxyphenyl)-3,5-heptanediol	[93]
*D. zingiberensis* rhizomes	Dyslipidemia	3T3-L1 cells	Inhibited the differentiation and lipid accumulation of 3T3-L1 cells	Diarylheptanoid	[94]
*D. alata* tubers	Dyslipidemia	Hyperlipidemic hamsters	Ameliorated lipid metabolism in association with gut microbiota modulation	Resistant starch	[95]
Chinese yams rhizomes	Hyperlipidemia Insulin resistance	Obesity-induced insulin resistance and hyperlipidemia in mice	Lowered the levels of LDL, cholesterol, leptin and IL-1β in serum, and down-regulated the expression of MMP-3 in visceral fat tissues	Polysaccharides	[96]
Shanghai Winherb Medical S & T Development (Shanghai, China)	Atherosclerosis	Ovariectomized ApoE-/- mice Human umbilical vein endothelial cells and Macrophages	Increased the level of ERα and eNOS protein Suppress TNFα expression Antiadipogenic effects	Pseudoprotodioscin	[97]
Chenguang biotechnology Co. Ltd., (baoji, China	Atherosclerosis	HFD-OVX-treated LDLR-/- mice	Inhibited postmenopausal Atherosclerosis via inhibiting oxidative stress, inflammation, apoptosis and promoting autophagy partly through PGC-1α/ERα pathway	Disocin	[98]
*D. nipponica* Makino rhizomes	Lipid disorder Atherosclerosis	High-fat diet-fed ApoE-/- mice	Reduce the levels of three major modifiable lipid risk factors, LDL-C, HDL-C, and TG Inhibited PCSK9/LDLR signaling pathway	-	[99]
*Dioscoreae* rhizomes	Hypercholesterolemia	Hypercholesterolemic rat models	Decreased body weight gain, liver weight ratio, serum lipids levels and hepatic lipids accumulation	-	[100]
*D. esculenta* tubers	Diabetes	Type 2 diabetes rat model	Increased muscle sex steroid hormone levels and decreased insulin resistance	Diosgenin	[101]
*D. opposita* Thunb	Diabetes	STZ-induced diabetic rats	Decreased the glucose and insulin levels and MDA contents	Polysaccharides	[102]
*D. nipponica* rhizomes	T2DM	Insulin-induced HepG2 cells Palmitic acid-induced AML12 cells High-fat diet- and streptozotocin-induced T2DM rats	Inhibited miR-125a-5p/STAT3 signaling pathway and alleviate glycolipid metabolic disorder	Dioscin	[103]
Shanghai Tauto Biochemical Technology Co., Ltd. (Shanghai, China)	NAFLD	Mice models of NAFLD	Alleviated liver lipid accumulation symptoms and improved the levels of serum and hepatic biochemical parameters	Dioscin	[104]
-	NAFLD	HFD-induced NAFLD rat	Ameliorated the hepatic lipid accumulation and HFD-induced liver function disturbance	Diosgenin	[105]
Beijing gersion Bio-Technology Co., Ltd. (Beijing, China)	NAFLD	High-fat diet-fed NAFLD rats	Suppressed excessive weight gain, reduced serum levels of total cholesterol and triglycerides, and decreased liver fat accumulation	Diosgenin	[106]

Abbreviations: HFD: high-fat-diet; CD36: cluster of differentiation 36; TNF-α: tumor necrosis factor-α; MCP-1: monocyte chemoattractant protein-1, IL-6: interleukin-6; LDL: low-density lipoprotein; MMP-3: matrix metalloproteinase-3; eNOS: endothelial nitric oxide synthase; Erα: estrogen receptor alpha; PGC-1α: peroxisome proliferator-activated receptor-γ coactivator 1-α; HDL-C; high-density lipoprotein cholesterol; TG: triglyceride; HFD-OVX: HFD and ovariectomy; LDLR-/-: low-density lipoprotein receptor deficient; PCSK9: proprotein convertase subtilisin/kexin type 9; STZ: streptozotocin; MDA: malondialdehyde; AML12: alpha mouse liver 12; T2DM: type 2 diabetes mellitus; NAFLD: non-alcoholic fatty liver disease.

## Data Availability

Not applicable.
